# Effects of Unloaded vs. Loaded Plyometrics on Speed and Power Performance of Elite Young Soccer Players

**DOI:** 10.3389/fphys.2017.00742

**Published:** 2017-09-26

**Authors:** Ronaldo Kobal, Lucas A. Pereira, Vinicius Zanetti, Rodrigo Ramirez-Campillo, Irineu Loturco

**Affiliations:** ^1^Nucleus of High Performance in Sport (NAR), São Paulo, Brazil; ^2^Red Bull Brazil, Jarinú, Brazil; ^3^Department of Physical Activity Sciences, Research Nucleus in Health, Physical Activity and Sport, University of Los Lagos, Osorno, Chile

**Keywords:** team-sports, football, power output, acceleration, youth athletes

## Abstract

The purpose of this study was to investigate the effects of loaded and unloaded plyometric training strategies on speed and power performance of elite young soccer players. Twenty-three under-17 male soccer players (age: 15.9 ± 1.2 years, height: 178.3 ± 8.1 cm, body-mass (BM): 68.1 ± 9.3 kg) from the same club took part in this study. The athletes were pair-matched in two training groups: loaded vertical and horizontal jumps using an haltere type handheld with a load of 8% of the athletes' body mass (LJ; *n* = 12) and unloaded vertical and horizontal plyometrics (UJ; *n* = 11). Sprinting speeds at 5-, 10-, and 20-m, mean propulsive power (MPP) relative to the players' BM in the jump squat exercise, and performance in the squat jump (SJ) and countermovement jump (CMJ) were assessed pre- and post-training period. During the experimental period, soccer players performed 12 plyometric training sessions across a 6-week preseason period. Magnitude based inferences and standardized differences were used for statistical analysis. A *very likely* increase in the vertical jumps was observed for the LJ group (99/01/00 and 98/02/00 for SJ and CMJ, respectively). In the UJ group a *likely* increase was observed for both vertical jumps (83/16/01 and 90/10/00, for SJ and CMJ, respectively). An *almost certainly* decrease in the sprinting velocities along the 20-m course were found in the LJ group (00/00/100 for all split distances tested). Meanwhile, in the UJ *likely* to *very likely* decreases were observed for all sprinting velocities tested (03/18/79, 01/13/86, and 00/04/96, for velocities in 5-, 10-, and 20-m, respectively). No meaningful differences were observed for the MPP in either training group (11/85/04 and 37/55/08 for LJ and UJ, respectively). In summary, under-17 professional soccer players increased jumping ability after a 6-week preseason training program, using loaded or unloaded jumps. Despite these positive adaptations, both plyometric strategies failed to produce worthwhile improvements in maximal speed and power performances, which is possible related to the interference of concurrent training effects. New training strategies should be developed to ensure adequate balance between power and endurance loads throughout short (and high-volume) soccer preseasons.

## Introduction

In prospective training programs, coaches and sports scientists face the challenge of developing multiple physical and technical capacities in young athletes. In this context, the proper development of speed-related abilities has a determinant role in improving athletic performance. More recently, in professional team sports, a progressive and gradual increase in the demands of maximal sprints and explosive game actions has been observed during training and official competitions (Barnes et al., 2014; Bush et al., 2015). For example, during an official soccer match, between 7 and 12% of the total distance is covered in high-speed running (>5 m.s^−1^), while from 1 to 4% is covered by sprinting activities (>7 m.s^−1^) (Bradley et al., 2009; Di Salvo et al., 2010). Importantly, these high-intensity tasks are frequently executed prior to decisive situations (e.g., goal scores) (Faude et al., 2012). As such, previous studies performed over seven consecutive seasons (from 2006 to 2013) in the English Premier League have demonstrated that professional soccer players have gradually become faster and capable of covering greater distances at higher speeds (from 9.12 to 9.55 m·s^−1^ for mean sprint velocity; and from 232 to 350 m for sprint distance, respectively) (Barnes et al., 2014; Bush et al., 2015). Thus, the development of neuromuscular abilities in soccer players has become crucial to cope with the progressive match velocity demands. This is especially important for young players initiating their elite careers, who need to focus on the gradual development of their physical abilities to successfully achieve professionalism (Ford et al., 2011; Williams, 2016).

Different training strategies have been effectively implemented to improve jumping and sprinting abilities in soccer players from different age categories (Garcia-Pinillos et al., 2014; Loturco et al., 2015a, 2016b; Silva et al., 2015). In this sense, despite their extensive use, the implementation of “traditional strength training” (i.e., heavy strength training) might be inappropriate when applied to young athletes, due to the limitations naturally imposed by the maturation process (Behm et al., 2008). Conversely, plyometric training seems to be a practical, safe, and efficient strategy for enhancing neuromuscular performance in young athletes (Loturco et al., 2015a; Ramirez-Campillo et al., 2015a,b). In fact, Loturco et al. (2015a) compared the effects of unloaded vertical vs. horizontal plyometrics on sprint performance of U-20 soccer players, revealing distinct performance adaptations in response to each specific training mode. As such, the horizontal jump group presented greater improvements in speed capacity over short distances (0–10 m), whereas the vertical jumps were superior to produce improvements over longer distances (10–20 m).

Alternatively, previous studies have investigated the effects of loaded jumps, with an haltere type handheld (Cronin et al., 2014; McKenzie et al., 2014; Rosas et al., 2016). Cronin et al. (2014) demonstrated acute improvement in jumping performance when using external overloading during plyometric jumps, which can be explained by the significant increase in ground reaction force and impulse promoted by the use of additional loads. More recently, Rosas et al. (2016) analyzed the effects of jumping with or without haltere type handheld loading on vertical and horizontal jump performances of youth soccer players, finding interesting and relevant results. After 6 weeks of training, both groups improved jump performances; however, the loaded jump group presented greater improvements in vertical and horizontal jump capacities. Nevertheless, it remains to be established whether this training strategy is also capable of increasing the maximal sprint capacity of young athletes. Therefore, the aim of this study was to investigate the effects of loaded and unloaded plyometric training strategies on speed and power performance of elite young soccer players.

## Materials and methods

### Participants

Twenty-three top-level under-17 male soccer players from the same club (age: 15.9 ± 1.2 years, height: 178.3 ± 8.1 cm, body-mass (BM): 68.1 ± 9.3 kg) took part in this study. The athletes were divided in two training groups: loaded vertical and horizontal jumps using an haltere type handheld with a load of 8% of the athletes' BM (McKenzie et al., 2014) (LJ; *n* = 12) and unloaded vertical and horizontal plyometrics (UJ; *n* = 11). Three players from the LJ group were excluded from the sample due to injuries unrelated to the proposed training/testing. Therefore, twenty players completed the study (*n* = 9 and *n* = 11 for LJ and UJ, respectively). The study protocol took place prior to the competitive season, during the preseason training period. The study was approved by the Anhanguera-Bandeirante University Ethics Committee and the participants and their legal guardians signed an informed consent form prior to research commencement.

### Experimental design

In this study, a parallel two-group, longitudinal design was conducted to test the effectiveness of two training programs on speed and power performance of elite soccer players. Players were pair-matched according to their baseline performance in the 20-m sprint test, and group allocation was performed by tossing a coin. All athletes had been previously familiarized with the performance tests. Sprinting speeds at 5-, 10-, and 20-m, mean propulsive power (MPP) relative to the players' BM in the jump squat exercise, and performance in the squat jump (SJ) and countermovement jump (CMJ) were assessed pre- and post-training period. Prior to all testing sessions, a general and specific warm-up routine was performed, involving light running (5-min at a self-selected pace) and submaximal attempts at each testing exercise (e.g., submaximal sprints and vertical jumps). During the experimental period, all soccer players performed 12 plyometric training sessions. A typical weekly training schedule and the detailed power-oriented training program across the 6-week preseason period are presented in Tables 1, 2.

**Table 1:** Typical weekly training schedule for the young soccer players.

	**Monday**	**Tuesday**	**Wednesday**	**Thursday**	**Friday**
Morning	Tec/Tac 60′	Tec/Tac 70′	Tec/Tac 80′	Tec/Tac 60′	Tec/Tac 60′
Afternoon	LJ/UJ 30′	Rest	LJ/UJ 30′	Rest	Tec/Tac 40′

*Tec/Tac, technical and tactical training based on specific technical actions (e.g., goal shooting, corner kick situations) and small-sided games of different formats; LJ, loaded vertical and horizontal jumps using an haltere type handheld with 8% of the players' body mass; UJ, unloaded vertical and horizontal plyometrics*.

**Table 2:** Training protocols for both groups over the 6-week training period.

**Week 1**	**Week 2**	**Weeks 3 and 4**	**Week 4**	**Week 6**
LJ or UJ (2 sessions/week)	LJ or UJ (2 sessions/week)	LJ or UJ (2 sessions/week)	LJ or UJ (2 sessions/week)	LJ or UJ (2 sessions/week)
2 × 6 VJ	3 × 6 VJ	4 × 6 VJ	6 × 6 VJ	4 × 6 VJ
2 × 6 HJ	3 × 6 HJ	4 × 6 HJ	6 × 6 HJ	4 × 6 HJ

*LJ, loaded vertical and horizontal jumps using an haltere type handheld with 8% of the players' body mass; UJ, unloaded vertical and horizontal plyometrics; VJ, vertical jump; HJ, horizontal jump*.

### Vertical jumping tests

Vertical jumping height was determined using both SJ and CMJ. In the SJ, subjects were required to remain in a static position with a 90° knee flexion angle for 2-s before jumping. In the CMJ, the soccer players were instructed to execute a downward movement followed by a complete extension of the legs. The SJ and CMJ were executed with the hands fixed on the hips. All jumps were performed on a contact platform (Elite Jump System® S2 Sports, São Paulo, Brazil) (Loturco et al., 2017b). The obtained flight time (t) was used to estimate the jump height (h) (i.e., *h* = gt^2^/8). A total of five attempts were allowed for each jump, interspersed by 15-s. The best attempts at SJ and CMJ were retained.

### Bar mean propulsive power in jump squat exercise

Bar maximum MPP in the jump squat exercise was assessed on a Smith machine (Hammer Strength, Rosemont, IL, USA). Players were instructed to execute two repetitions at maximal intensity for each load, starting at 40% of their BM. Athletes executed a knee flexion until the thigh was parallel to the ground (≈100° knee angle) and, after a command, jumped as fast as possible without losing contact between their shoulder and the bar. A load of 10% BM was gradually added until a decrease in MPP was observed. A 5-min interval between sets was provided. To determine MPP, a linear transducer (T-Force, Dynamic Measurement System; Ergotech Consulting S.L., Murcia, Spain) was attached to the Smith machine bar. The technical specification of the MPP analysis and its calculation have been previously described (Sanchez-Medina et al., 2010; Loturco et al., 2015c,d, 2016b, 2017a). The maximum MPP value relativized by the players' BM was retained for data analysis purposes.

### Sprinting speed

Four pairs of wireless single-beam light gates (Smart Speed System, Fusion Equipment, AUS) were positioned at distances of 0, 5-, 10-, and 20-m along the sprinting course. The soccer players sprinted twice, starting from a standing position 0.3 m behind the starting line. To avoid weather influences, the sprint tests were performed on an indoor running track. A 5-min rest interval was allowed between the two attempts and the fastest time was considered for the analyses. The average speeds from zero to the respective gates (5-, 10-, and 20-m) were considered for data analysis purposes.

### Statistical analysis

Data are presented as mean ± standard deviation (SD). To analyze the differences in the vertical jumps, sprinting velocities, and MPP in the LJ and UJ groups, pre- and post-training, the differences based on magnitudes were calculated (Batterham and Hopkins, 2006). The magnitude of the within-group changes in the different performance variables, or between-group differences in the changes, were expressed as standardized mean differences (Cohen's *d*). The smallest worthwhile change was set by using the Cohen's principles for a small effect size (ES: 0.2) for each variable tested (Cohen, 1988). The quantitative chances of finding differences in the variables tested were assessed qualitatively as follows: <1%, almost certainly not; 1–5%, very unlikely; 5–25%, unlikely; 25–75%, possible; 75–95%, likely; 95–99%, very likely; >99%, almost certain. A meaningful difference was considered using the mechanistic inference, based on threshold chances of 5% for substantial magnitudes (Hopkins et al., 2009). Therefore, if the chances of having better and poorer results were both >5%, the true difference was assessed as unclear. Additionally, the magnitudes of the standardized differences were interpreted using the following thresholds: <0.2, 0.2–0.6, 0.6–1.2, 1.2–2.0, 2.0–4.0, and >4.0 for trivial, small, moderate, large, very large, and near perfect, respectively (Hopkins et al., 2009). All performance tests used herein demonstrated small errors of measurement, as presented by their high levels of accuracy and reproducibility (CV < 5% and ICC > 0.90 for all assessments) (Hopkins et al., 2009).

## Results

**Figure 1:**
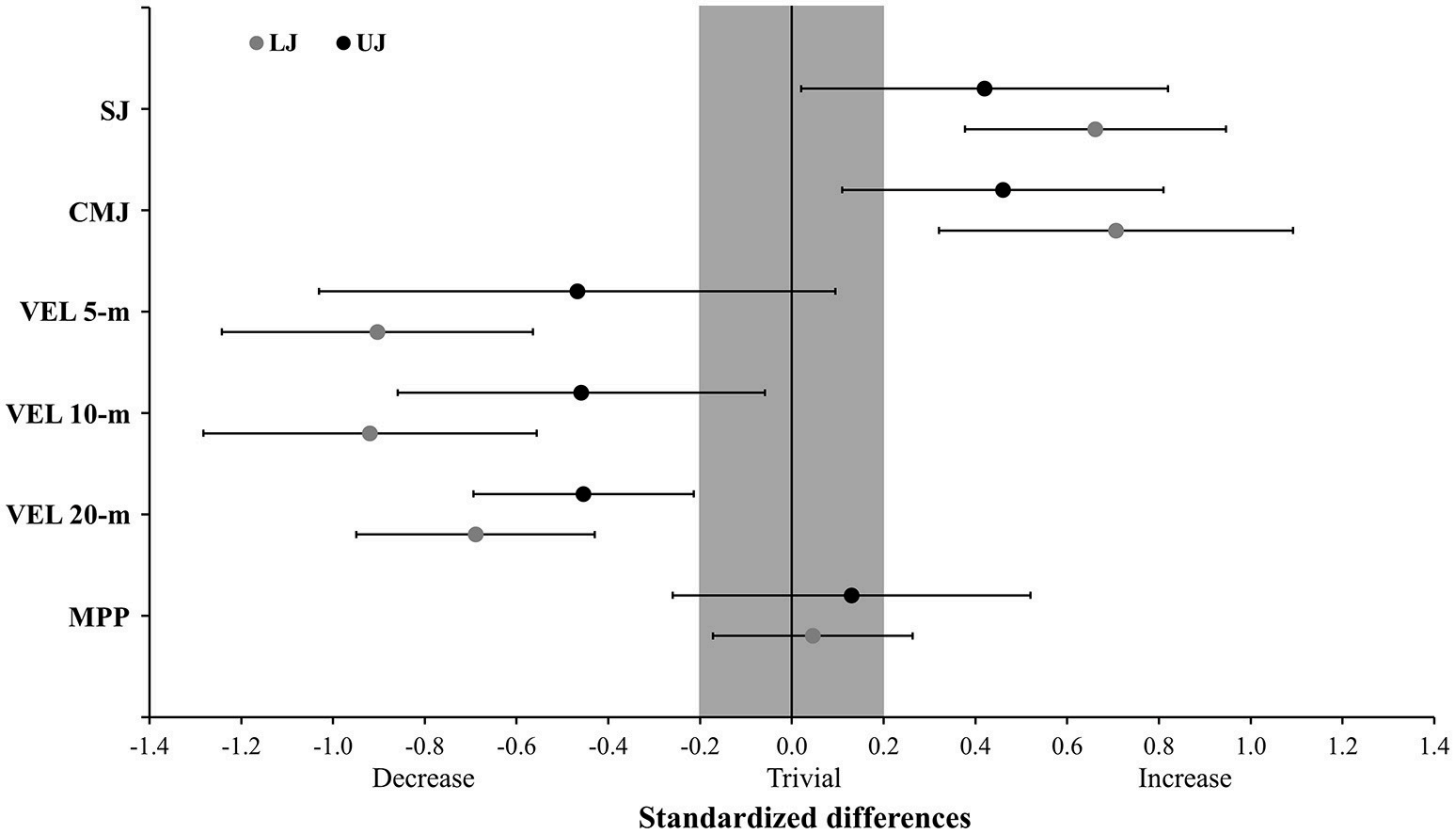
Standardized differences of the performance tests comparing pre- and post- the 6-week preseason period in young soccer players. LJ, loaded jump group; UJ, unloaded jump group; SJ, squat jump; CMJ, countermovement jump; VEL, velocity; MPP, maximum mean propulsive power relative to the players' body mass; error bars represent 90% confidence limits; gray area represents the smallest worthwhile change based on Cohen's units (effect size > 0.2).

Figure 1 depicts the standardized differences between pre- and post-assessments for both LJ and UJ groups of the plyometric-oriented training program. A *very likely* increase in the vertical jumps was observed for the LJ group. In the UJ group a *likely* increase was observed for both vertical jumps. An *almost certainly* decrease in the sprinting velocities along the 20-m course was found in the LJ group. Meanwhile, in the UJ *likely* to *very likely* decreases were observed for all sprinting velocities tested. No meaningful differences were observed for the MPP in either training group. Table 3 shows the comparisons between the changes observed for LJ compared with those found for UJ.

**Table 3:** Comparisons of the changes observed for both groups of training in the performance tests after a 6-week preseason period in young soccer players.

	**Loaded jump**		**Unloaded jump**		**Changes observed for LJ compared with UJ**	
	**Pre**	**Post**	**Pre**	**Post**	**Standardized differences (90% CL)**	**Percent chances of better/trivial/poorer effect**
SJ (cm)	36.6 ± 5.2	40.4 ± 4.5	39.2 ± 4.4	41.1 ± 2.9	0.43 (−0.11; 0.96) *small*	76/21/03 *likely*
CMJ (cm)	38.2 ± 4.5	41.7 ± 4.4	40.3 ± 4.4	42.4 ± 3.9	0.33 (−0.21; 0.87) *small*	67/28/05 *possibly*
VEL 5-m (m·s^−1^)	4.93 ± 0.25	4.68 ± 0.29	4.79 ± 0.18	4.69 ± 0.23	0.65 (0.08; 1.21) *moderate*	01/08/91 *likely*
VEL 10-m (m·s^−1^)	5.75 ± 0.20	5.55 ± 0.24	5.66 ± 0.21	5.55 ± 0.20	−0.45 (−0.98; 0.08) *small*	02/19/79 *likely*
VEL 20-m (m·s^−1^)	6.70 ± 0.23	6.53 ± 0.23	6.60 ± 0.23	6.49 ± 0.24	−0.27 (−0.62; 0.09) *small*	01/36/63 *possibly*
MPP (W·kg^−1^)	9.51 ± 1.48	9.58 ± 1.35	9.20 ± 1.14	9.36 ± 1.12	−0.06 (-0.38; 0.51) *trivial*	16/30/54 *unclear*

*SJ, squat jump; CMJ, countermovement jump; VEL, velocity; MPP, mean propulsive power relative to the players' body mass in the jump squat exercise; CL, confidence limits*.

## Discussion

This study aimed to compare the effects of 6-weeks of loaded vs. unloaded plyometric training regimens on neuromuscular abilities of elite young soccer players. The main findings reported here are that: (1) both training strategies could meaningfully improve the vertical jumping ability of these athletes, and (2) independent of the training mode, the soccer players presented considerable impairments in their acceleration and speed capabilities. These outcomes have important implications in the practical field.

To some extent, our findings are in accordance with previous studies which have already reported positive effects of loaded and unloaded plyometric training programs on jumping ability of young soccer athletes (Loturco et al., 2015a; Ramirez-Campillo et al., 2015a,b). For example, Loturco et al. (2015a) observed meaningful (and specific) increases in vertical and horizontal jump performances of elite U-20 players who executed short-term training programs (3-week) exclusively composed of vertical or horizontal plyometrics. Likewise, with a design more similar to the one used in the present investigation, Rosas et al. (2016) showed a superior capacity of loaded jumps (in comparison with unloaded jumps) to induce functional gains (i.e., kicking velocity and jumping ability) in 63 male youth soccer athletes. The same holds true for our study: the group who trained under loaded conditions reported higher increases in CMJ and SJ heights than the UG (Table 3). The reasons behind the apparent superiority of the LG could be related to the overload principle, which states that muscles must be stressed beyond their present capacity to stimulate an adaptive response (Carlson, 2004; Issurin, 2013). Therefore, besides the chronic responses normally expected from a regular plyometric training regimen (e.g., enhanced jump coordination and stretch-shortening cycle efficiency; de Villarreal et al., 2009), it may be speculated that the use of handheld loads enabled players to apply greater amounts of force against the ground in the direction of the intended movement (vertical or horizontal axes) over a longer time period. This mechanical adjustment possibly generates higher impulses (as an “extra overload”) during the jumps (Cronin et al., 2014), thus producing superior adaptations in jumping ability in the LG.

Although a previous research has already investigated the effects of handheld loading on neuromechanical capacities of young soccer players (Rosas et al., 2016), this is the first study to analyze its impacts on speed ability. Remarkably, despite the strong correlations already found between jump and sprint performances (Cronin and Hansen, 2005; Loturco et al., 2015b), neither the UG nor LG presented meaningful increases in maximal running speed. Indeed, the proper development of acceleration and speed capacities in elite soccer players throughout age categories and successive training seasons has been demonstrated to be relatively problematic in the literature (Loturco et al., 2015c; Kobal et al., 2016). Partially, it could be elucidated by analyzing the progressive increase in the typical endurance loads that gradually occurs throughout the prospective development of soccer athletes. In fact, due to the congested fixture schedule of elite soccer—which includes high volumes of “predominantly aerobic activities” (i.e., specific soccer training) and relatively low volumes of strength-power training—the players are continuously exposed to concurrent training effects. These interference effects seem to be yet more pronounced during preseasons, where the players usually have to perform a great number of technical and tactical sessions in a short-time period (3–6 weeks) without an appropriate recovery between sessions (Coutts et al., 2007; Coutts and Reaburn, 2008; di Fronso et al., 2013). Also in this study, it is probable that the young players had some difficulties coping with the high demand of aerobic loads throughout the congested preseason period (Table 1), thus presenting impaired speed ability after the training intervention. Therefore, even with the substantial increases reported in vertical and horizontal jump capacities, the soccer players were incapable of sprinting faster. It is reasonable to believe that the neuromuscular stimuli experienced by our youth players was incapable of promoting a positive transference effect between jump performance gains and sprinting speed, as previously reported in the literature (Loturco et al., 2015a, 2016b).

In the same way, for both groups of training the MPP did not increase after the preseason period. This is partially in contrast with a previous study performed in our sports laboratory, which demonstrated meaningful improvements in relative values of muscle power in under-20 soccer players who performed loaded jumps (i.e., jump squats) during an inter-season training period (Loturco et al., 2016b). The lack of improvement in power production observed herein can be explained in different forms. As reported before, the high volume of soccer specific training during preseasons may lead to interference effects from this aerobically predominant training strategy over the specific neuromechanical adaptations. Furthermore, to be effective in improving speed and power abilities, a neuromuscular training strategy for top-level soccer players seems to rely on two important points: reducing the volume of technical and tactical training (Loturco et al., 2016b); and improving the intensity and volume of neuromuscular training (Silva et al., 2015; Loturco et al., 2016a,b). For instance, large improvements in the sprinting speed and in the MPP were observed after an inter-season period of elite soccer players where only low volumes of small sided-games were programmed during the experimental intervention (Loturco et al., 2016b). This respective period comprised resistance training using loaded jump squat performed at the optimum power loads (i.e., workloads able to maximize power output) where athletes trained with loads corresponding to ~60% of their BM (Loturco et al., 2016b) vs. the load of 8% of the players' BM used in the present study. That said, it is worth highlighting that coaches and sport scientists who are interested in maximizing the speed and power abilities of young athletes pay attention not only to the specific content of neuromuscular training approaches, but also to the total volume of specific soccer sessions executed by the players (Silva et al., 2015; Loturco et al., 2016b). Without an adequate balance between these distinct physical and specific technical-tactical strategies, the proper development of maximal running speed throughout the age categories could be significantly affected (Kobal et al., 2016; Nakamura et al., 2016), compromising the prospective development of elite soccer players.

Accordingly, it has been already shown that the sprinting speed and MPP of professional adult soccer players were not different from their younger counterparts (Loturco et al., 2014; Kobal et al., 2016). Meanwhile, performance in the Yo-Yo intermittent recovery test increased progressively from under-17 to adult age categories (Kobal et al., 2016). This reinforces the notion that since the early stages of development, soccer training programs are focused on the promotion of specific match activities as well as the aerobic capacity, with less importance being paid to neuromuscular abilities, which has an important and pivotal impact on the physical development of these athletes for the older categories, where the physical demands are higher and players need to deal with the competitiveness to achieve success in important professional teams.

According to numerous studies conducted with top-level athletes (Buchheit et al., 2010; Campos-Vazquez et al., 2015; Iacono et al., 2015; Loturco et al., 2016b), this investigation is limited by the absence of a control group (i.e., soccer players maintaining their regular training routine without adding plyometric exercises), the small sample sizes and the inevitable and expected dropouts (3 subjects in the LG and 1 subject in the UG). Even with these limitations, we reported worthwhile improvements in the jumping ability of elite young soccer athletes in response to two different applied plyometric training strategies (i.e., handheld loaded group vs. unloaded group). Lastly, it is worth noting that meaningful increases in neuromechanical capacities of top-level players is not a *commonplace* occurrence in investigations performed during short and high-volume soccer preseasons (Taylor et al., 2012; Meckel et al., 2014; Loturco et al., 2015c).

To conclude, under-17 top-level soccer players could improve their jump performance after a 6-week preseason training program using loaded and unloaded jumps. Nevertheless, this improvement was not accompanied by meaningful increases in maximal sprinting speed and relative muscle power. Therefore, it is strongly recommended for future studies to better manage specific soccer training content (frequency, volume and intensity) and assess different loading and exercise strategies, to provide sufficient stimulus to increase the different spectrum of neuromuscular abilities in elite youth soccer players. In this respect, it should be emphasized that there is an emergent need to produce faster (and more efficient) players to cope with the increased physical and technical demands of modern soccer.

## Author contributions

Designed the work: IL; data acquisition: RK, LP, VZ, and IL; analysis and interpretation of data: LP, RR, and IL; drafting the work: RK, LP, and IL; revising critically the work: LP, VZ, RR, and IL final approval of the version to be published: RK, LP, VZ, RR, and IL; agree to be accountable for all aspects of the work in ensuring that questions related to the accuracy or integrity of any part of the work were appropriately investigated and resolved: RK, LP, VZ, RR, and IL.

### Conflict of interest statement

The authors declare that the research was conducted in the absence of any commercial or financial relationships that could be construed as a potential conflict of interest.
